# Stimulation of Alpha_1_-Adrenergic Receptor Ameliorates Cellular Functions of Multiorgans beyond Vasomotion through PPAR*δ*

**DOI:** 10.1155/2020/3785137

**Published:** 2020-02-01

**Authors:** Yong-Jik Lee, Hyun Soo Kim, Hong Seog Seo, Jin Oh Na, You-Na Jang, Yoon-Mi Han, Hyun-Min Kim

**Affiliations:** ^1^Cardiovascular Center, Division of Cardiology, Department of Internal Medicine, Korea University Guro Hospital, Korea University College of Medicine, Seoul 08308, Republic of Korea; ^2^Department of Anatomy, Korea University College of Medicine, Seoul 08308, Republic of Korea

## Abstract

Cells can shift their metabolism between glycolysis and oxidative phosphorylation to enact their cell fate program in response to external signals. Widely distributed *α*_1_-adrenergic receptors (ARs) are physiologically stimulated during exercise, were reported to associate with the activating energetic AMPK pathway, and are expected to have biological effects beyond their hemodynamic effects. To investigate the effects and mechanism of AR stimulation on the physiology of the whole body, various *in vitro* and *in vivo* experiments were conducted using the AR agonist midodrine, 2-amino-*N*-[2-(2,5-dimethoxyphenyl)-2-hydroxy-ethyl]-acetamide. The expression of various biomarkers involved in ATP production was estimated through Western blotting, reverse transcription polymerase chain reaction, oxygen consumption rate, enzyme-linked immunosorbent assay (ELISA), fluorescence staining, and Oil red O staining in several cell lines (skeletal muscle, cardiac muscle, liver, macrophage, vascular endothelial, and adipose cells). In spontaneously hypertensive rats, blood pressure, blood analysis, organ-specific biomarkers, and general biomolecules related to ATP production were measured with Western blot analysis, immunohistochemistry, ELISA, and echocardiography. Pharmacological activation of *α*_1_-adrenergic receptors in C2C12 skeletal muscle cells promoted mitochondrial oxidative phosphorylation and ATP production by increasing the expression of catabolic molecules, including PPAR*δ*, AMPK, and PGC-1*α*, through cytosolic calcium signaling and increased GLUT4 expression, as seen in exercise. It also activated those energetic molecules and mitochondrial oxidative phosphorylation with cardiomyocytes, endothelial cells, adipocytes, macrophages, and hepatic cells and affected their relevant cell-specific biological functions. All of those effects occurred around 3 h (and peaked 6 h) after midodrine treatment. In spontaneously hypertensive rats, *α*_1_-adrenergic receptor stimulation affected mitochondrial oxidative phosphorylation and ATP production by activating PPAR*δ*, AMPK, and PGC-1*α* and the relevant biologic functions of multiple organs, suggesting organ crosstalk. The treatment lowered blood pressure, fat and body weight, cholesterol levels, and inflammatory activity; increased ATP content and insulin sensitivity in skeletal muscles; and increased cardiac contractile function without exercise training. These results suggest that the activation of *α*_1_-adrenergic receptor stimulates energetic reprogramming via PPAR*δ* that increases mitochondrial oxidative phosphorylation and has healthy and organ-specific biological effects in multiple organs, including skeletal muscle, beyond its vasomotion effect. In addition, the action mechanism of *α*_1_-adrenergic receptor may be mainly exerted via PPAR*δ*.

## 1. Introduction

In the life of an organism, cellular bioenergetic needs are supplied through interconnected glycolysis in the cytoplasm and the tricarboxylic acid (TCA) cycle and oxidative phosphorylation in the mitochondria. In the sequential reactions of glycolysis, glucose is metabolized to pyruvate, liberating pyruvate, adenosine triphosphate (ATP), and NADH in the cytoplasm. Under normoxic conditions, pyruvate enters the mitochondria and is oxidized to CO_2_ and H_2_O via the TCA cycle, which generates NADH that is oxidized by oxidative phosphorylation reactions to generate a large amount of ATP and NAD^+^ in the mitochondria. In macromolecule synthesis, glucose can serve the pentose phosphate pathway for RNA and DNA synthesis and NADPH for fatty acid synthesis. Upregulation of glycolysis is a critical step in increasing the flux to synthesize macromolecules. The TCA cycle also provides substrates for the synthesis of isoprenoids, cholesterol, flavonoids, and fatty acids and for the posttranslational acetylation of histones and proteins [1]. These biosynthetic reactions require continual cataplerotic extraction of intermediates from glycolysis and the TCA cycle, and thus, they maintain these essential metabolic pathways [2]. Glycolysis is maintained by increasing the metabolic flux through the glycolytic pathway, but to replenish the TCA cycle, two anapleroticmolecules, oxaloacetate and glutamate, are used. In activated cells producing biomass, neither glucose nor glutamine is fully oxidized to generate ATP [3], because the anaplerotic flux must be greater than the cataplerotic flux to replenish the intermediates of the TCA cycle. Therefore, excess flux through those pathways is maintained by reducing pyruvate to lactate, which generates the necessary NAD^+^ needed to sustain both glycolysis and the TCA cycle, even under aerobic conditions. Thus, glycolysis is critical to cellular functions (such as inflammation and cell proliferation) that require macromolecules [4].

Recent studies have shown that how cells produce energy crucially affects the function of cells and organs during exercise and immune reactions. Cells can shift their metabolism between glycolysis and oxidative phosphorylation to enact a functional fate program in response to an external signal. For example, the preferential reliance of classically activated M1-type macrophages on glycolysis and alternatively activated M2-type macrophages on oxidative metabolism [4] is similar to the fuel preferences of fast-twitch (type II) and slow-twitch (type I) skeletal muscle fibers [5]. This paradigm of coupling a cell's metabolism to its functions has also been studied in heart and tissue-specific stem cells, where metabolic switching between oxidative metabolism and glycolysis controls heart function and hematopoietic stem cell activation, respectively [6, 7].

In clinical situations, the mitochondrial function of ATP production is influenced by factors such as age, sex, nutritional status, and exercise performance, and we suggest that those factors program bioenergetic metabolism to be glycolysis or oxidative phosphorylation, which changes the metabolic function of multiple organs throughout the body. In obesity and physical inactivity, which are frequently associated with mitochondrial dysfunction [10], accelerated glycolysis is expected to match the degree of mitochondrial dysfunction in oxidative phosphorylation, consequently affecting the functions of organs throughout the body. This pathophysiology could explain why metabolic syndrome consists of a cluster of metabolic conditions, such as hypertriglyceridemia, hyper-low-density lipoprotein (LDL) cholesterol, hypo-high-density lipoprotein (HDL) cholesterol, insulin resistance, abnormal glucose tolerance, hypertension, vascular inflammation, atherosclerosis, and renal, liver, and heart diseases [11]. In this context, exercise is a way to modulate the metabolic pathway of ATP production and has a healthy effect by activating catabolic molecules such as PPAR*δ*, AMPK, and PGC-1*α*, which stimulate mitochondrial oxidative phosphorylation [8] to change the plasticity of skeletal muscle.

The sympathetic nervous system is an integrated network that can simultaneously produce various effects on all organs in the body to maintain homeostasis in a changing environment. Its physiological and metabolic responses are mediated through the action of endogenous catecholamine, norepinephrine, and epinephrine on adrenergic receptors. Both the *α*_1_-adrenergic receptor (AR) and *β*-AR are activated during exercise, and *α*_1_-AR contributes deeply to hemodynamic support during exercise. Unlike *β*-AR, *α*_1_-AR has been reported to have beneficial health effects even when chronically stimulated. It has a protective role in cardiac contractile function [12–14] that differs from the complex effects of *β*-AR [15]. Moreover, it is associated with energy expenditure in skeletal muscle metabolism through its activation of AMPK [16]. Interestingly, *α*_1_-AR is distributed not only in the blood vessels of muscles but also ubiquitously in most organs, including skeletal muscle fibers [16, 17], the heart [18], adipose tissue [19], the liver [20], and other organs [21], suggesting that it is distributed to those organs through the network of sympathetic nerves. Because *α*_1_-AR can be physiologically stimulated during exercise; is associated with the activation of AMPK [16], which stimulates ATP production through mitochondrial oxidative phosphorylation [8]; and is distributed widely throughout the body, we hypothesize that *α*_1_-AR stimulation could control the metabolic functional changes of all cells and organs with *α*_1_-ARs by coupling a cell's fueling metabolism to functional changes, as seen in exercise.

For this study, we performed cell experiments to investigate whether *α*_1_-AR stimulation promotes the expression of mitochondrial energetic molecules and oxidative phosphorylation processes and whether it affects the relevant biological functions of skeletal muscle cells, as seen in exercise. In addition, to determine whether *α*_1_-AR has the same metabolic functions in cells derived from multiple organs, we performed experiments with cardiomyocytes, endothelial cells, adipocytes, macrophages, and hepatic cells. We also used an animal model of metabolic syndrome without exercise training to investigate whether pharmacological *α*_1_-AR stimulation promotes the expression of genes for mitochondrial oxidative phosphorylation and causes biologic changes in multiple organs.

## 2. Materials and Methods

### 2.1. Materials

L6 rat skeletal muscle, C2C12 mouse skeletal muscle, HL1 and H9C2 cardiac muscle, HUVEC human umbilical vein endothelial cell line, RAW 264.7 macrophages, and 3T3-L1 mouse preadipocyte cells were purchased from a Korean cell line bank (Seoul, Korea). Fetal bovine serum (FBS), antibiotic-antimycotic (AA) solution, and media were purchased from WELGENE Inc. (Daegu, Korea). TRIzol® reagent was purchased from Invitrogen (Carlsbad, CA, USA). PRO-PREP™ protein extraction solution and prestained protein size markers were purchased from iNtRON Biotechnology. Anti-AMPK*α* (*α* subunit total form) and anti-phospho-AMPK*α* (phosphorylated at Thr172) primary antibodies were purchased from Cell Signaling Technology, Inc. (Danvers, MA, USA). Primary antibodies for anti-PGC-1*α*, anti-PPAR*δ*, anti-mannose receptor, and hexokinase II and a rat adiponectin enzyme-linked immunosorbent assay (ELISA) kit were purchased from Abcam (Cambridge, UK). Primary antibodies for anti-cytochrome c oxidase (cytochrome c oxidase subunit 4 (COX4)), anti-glucose transporter 4 (GLUT4), anti-rabbit secondary antibody, and anti-mouse secondary antibody were acquired from Santa Cruz Biotechnology (Santa Cruz, CA, USA). Anti-alpha_1_-adrenergic receptor 1 was purchased from Novus (Littleton, CO, USA). ELISA kits for ATP, PPAR*δ*, AMPK, PGC-1*α*, interleukin- (IL-) 1*β*, IL-6, tumor necrosis factor-alpha (TNF-*α*), and reactive oxygen species (ROS) were purchased from MyBioSource (CA, USA). A Clarity™ Western ECL Substrate Kit was purchased from Bio-Rad (Hercules, CA, USA). X-ray film was obtained from Agfa (Mortsel, Belgium, Germany), and the developer and fixer reagents were purchased from Kodak (Rochester, NY, USA). Midodrine, compound C, sodium succinate, nitroblue tetrazolium, potassium phosphate monobasic, sodium phosphate dibasic, protease inhibitor cocktail, and Oil Red O reagent were purchased from Sigma-Aldrich (St. Louis, MO, USA). Immunohistochemistry reagents were purchased from Vector Laboratories (Burlingame, CA, USA).

### 2.2. Animal Preparation

Twenty-four male spontaneously hypertensive rats (SHRs) (3 weeks of age) were maintained under standardized conditions (21°C, 41–62% humidity) with a regular day/night (10/14 h) cycle and free access to water and a laboratory diet. All animal experiments were performed in compliance with the Korea University Animal Science Rules and Regulations (KUIACUC-2012-100). Experimental procedures and housing conditions were approved by the Committee of Animal Experimentation, Korea University. After 1 week of acclimation, we assigned the rats to 4 groups (6 rats per group) as follows: basal control (sacrificed at 4 weeks of age), group I (midodrine administration for 4 weeks, starting at 4 weeks of age; sacrificed at 8 weeks of age), group II (atenolol administration for 4 weeks, starting at 4 weeks of age; sacrificed at 8 weeks of age), and group III (drinking water only controls; sacrificed at 8 weeks of age). Basal control and group III received standard maintenance chow diets (K-H4 pellets, Ssniff; Soest, Germany) without any drugs; group I received the same diet with midodrine in the drinking water (0.3 mg/kg/day); and group II received the same diet with atenolol in the drinking water (1 mg/kg/day). Blood pressure (BP) was measured by tail-cuff plethysmography every 7 days using a BP-2000 Blood Pressure Analysis System™ (Visitech Systems Inc.; Apex, NC, USA). The animals underwent no exercise training. Animals in basal control were euthanized at 4 weeks of age, and the rats in the other groups were euthanized after 4 weeks of treatment at 8 weeks of age. Blood samples were obtained from the inferior vena cava, and the heart, aorta, liver, skeletal muscles, and visceral fat were dissected cleanly and weighed. Harvested organs were stored at - 80°C or in 10% (*w*/*v*) formalin for immersion fixation.

### 2.3. Cell Culture

HL1 cells were cultured in Claycomb medium containing norepinephrine (100 *μ*M) and maintained in a 37°C, 5% CO_2_ incubator. L6, C2C12, RAW 264.7, HL1, and H9C2 cells were cultured in DMEM containing 10% FBS and 1% AA solution and maintained in a 37°C, 5% CO_2_ incubator. The medium was replaced with new DMEM or Claycomb medium every 48–72 h. Cells were plated at a density of 1 × 10^4^ cells per well in 96-well culture dishes or 2 × 10^5^ cells per well in a six-well culture plate in Claycomb or DMEM medium containing 10% FBS and 1% antibiotic-antimycotic solution. Cells were cultured for 24–48 h and maintained in a 37°C, 5% CO_2_ incubator, and then, the medium was changed to Claycomb or DMEM containing 1% FBS. Thereafter, the cells were treated with midodrine (1–100 *μ*M) for 1–24 h. 3T3-L1 preadipocytes between 10 and 20 passages were plated at a density of 5 × 10^4^ cells per well in 24-well culture dishes in DMEM containing 10% calf serum and 1% antibiotic-antimycotic solution. When the 3T3-L1 cells reached confluency, differentiation medium was added to the cells along with midodrine (30 *μ*M) and GSK0660 (50 *μ*M), a PPAR*δ* antagonist. The differentiation medium contained 0.0125 mM dexamethasone, 12.5 mM 3-isobutyl-1-methylxanthine, 10 *μ*g/mL insulin, and 10% FBS. After a differentiation phase of two days, the medium was changed to one containing 10 *μ*g/mL insulin and 10% FBS. After incubation in the insulin medium for 2 days, the medium was changed to a maintenance medium containing 10% FBS.

### 2.4. Echocardiographic Examination of the Heart

After anesthetizing the rats by an intramuscular injection of Zoletil® (8 mg/kg) and xylazine (2 mg/kg), we obtained M-mode echo images with each rat lying on its left side. All examinations were performed using a Vivid 7 (GE Medical Systems; Milwaukee, WI, USA) with a 12 MHz transducer. After obtaining an optimal 2-dimensional short-axis view of the left ventricle at the level of the papillary muscles, M-mode tracings were recorded at a speed of 100 mm/s with simultaneous electrocardiogram recording. Cardiac wall thickness, dimensions, and mass were measured using a modification of the American Society of Echocardiography method from at least three consecutive cardiac cycles on the M-mode tracings [22].

### 2.5. Enzyme Activity Assay for Succinate Dehydrogenase (SDH)

Tissue samples were homogenized in phosphate-buffered saline (PBS) containing a 1% (*v*/*v*) protease inhibitor. The homogenized extracts were centrifuged at 13000 rpm and 4°C for 5 min, and then, the supernatants were transferred to new tubes. The incubation solution consisted of 1 M phosphate buffer (25 *μ*L), 0.2 M sodium succinate (125 *μ*L), 10 mg/mL nitroblue tetrazolium (25 *μ*L), and 235 *μ*L of distilled water per reaction, and samples were incubated for 20 min in a 37°C chamber. Enzyme solution (90 *μ*L) was added to the prewarmed incubation solution (410 *μ*L), and the reaction mixture was incubated for 30 min in a 37°C chamber. After termination of the reaction, the absorbance of the reaction mixture was measured at 550 nm. Enzyme activity was calculated using the following formula:

(1) $$\begin{array}{c} \text{Enzyme activity}=\frac{\text{absorbance of the enzyme reaction}–\text{absorbance of the diluted enzyme solution}}{\text{quantified protein amount}} \end{array}$$

### 2.6. Measurement of Blood Lipid Biochemistry and the Levels of ATP, ROS, IL-1*β*, TNF-*α*, Adiponectin, ROS, PPAR*δ*, AMPK, and PGC-1*α* (ELISA)

The total concentrations of cholesterol, HDL cholesterol, triglycerides, and LDL cholesterol in serum samples were measured on a Toshiba TBA-2000FR (Toshiba Medical Systems Corporation, Tochigi, Japan) according to the manufacturer's instructions in the Department of Laboratory Medicine (Diagnostic Tests), Korea University, Guro Hospital (Seoul, Korea). The levels of ATP, ROS, IL-1*β*, TNF-*α*, adiponectin, ROS, PPAR*δ*, AMPK, and PGC-1*α* in serum, tissue samples, or cell extracts were estimated according to the manufacturer's method.

### 2.7. Seahorse XF Analyzer Protocol

Oxygen consumption rate (OCR) analyses in C2C12 and H9C2 cells were completed using a Seahorse XFp system (Agilent, Santa Clara, CA, USA) according to the manufacturer's protocol. C2C12 cells were plated at 1 × 10^4^ cells per well, and H9C2 cells were plated at 1.5 × 10^3^ cells per well. After the cells settled, midodrine was added to the medium, and the cells were incubated for 24 h in a 37°C, 5% CO_2_ incubator. A sensor cartridge+utility plate containing calibrant was incubated overnight in a CO_2_-free incubator at 37°C. On the day of the analysis, assay medium similar to the culture medium was prepared (C2C12: 5.6 mM glucose and 4 mM L-glutamine; H9C2: 25 mM glucose and 4 mM L-glutamine), and the pH was adjusted to 7.4. The XFp miniplate was washed twice with assay medium, and assay medium (a final volume of 180 *μ*L) was added to the cells. Then, the XFp miniplate was equilibrated in a CO_2_-free incubator at 37°C for 60 min prior to assay initiation. Oligomycin, carbonyl cyanide-4-(trifluoromethoxy)phenylhydrazone, and antimycin A/rotenone were separately injected in each drug port of the sensor cartridge+utility plate and incubated in a CO_2_-free incubator for 10 min, and then, the OCR was measured.

### 2.8. Reverse Transcription Polymerase Chain Reaction

Total RNA was extracted using TRIzol® reagent according to the manufacturer's instructions. Complementary DNA was synthesized from total RNA using a Power cDNA Synthesis Kit, and polymerase chain reactions for PPAR*δ*, AMPK*α*_1_, mannose receptor, and *β*-actin were performed using a PCR PreMix Kit. The reaction mixture containing cDNA was preheated for 5 min at 95°C for the initial denaturation step. The polymerase chain reaction consisted of a denaturation step for 20 s at 95°C, an annealing step for 10 s at 55°C, an extension step for 30 s at 72°C, and a final extension step for 5 min at 72°C. The qPCR was performed using the following mouse primers: PPAR*δ* sense (5′-GGC AGA GTT GCT AGG GTT CC-3′) and antisense (5′-CAA GGA ACA CCC CAA GAC CT-3′), AMPK*α*_1_ sense (5′-CCT GCT TGA TGC ACA CAT GA-3′) and antisense (5′-TCA TCA AAA GGG AGG GTT CC-3′), and mannose receptor sense (5′-CGG CAT GGG TTT TAC TGC TA-3′) and antisense (5′-TAA ACT GCA CCT GCT CGT CC-3′). The experiments were performed using three independent biological replicates. Gene expression was normalized to the mRNA expression level of *β*-actin as an endogenous control, and fold changes in expression were calculated between treated and untreated control samples.

### 2.9. Western Blot Analysis

Protein amounts were estimated using the Bradford method. Extracted proteins (20–30 *μ*g) were loaded onto 10% (*v*/*v*) SDS-PAGE gels. Western blot analyses were performed using primary antibodies against *α*_1_-AR, AMPK*α*, p-AMPK*α*, PPAR*δ*, PGC-1*α*, mannose receptor, and hexokinase II. The dilution ratios of the primary antibodies for PPAR*δ*, AMPK, p-AMPK (at Thr172), PGC-1*α*, mannose receptor, and hexokinase II were 1 : 1000, and the dilution conditions for the secondary antibodies were as follows: anti-rabbit IgG antibodies for PPAR*δ*, AMPK, p-AMPK, PGC-1*α*, and mannose receptor were 1 : 5000 and anti-mouse IgG antibodies for *β*-actin and hexokinase II were 1 : 5000. Images were taken manually using Kodak GBX developer and fixer reagents.

### 2.10. Intracellular Calcium Measurement

The Ca^2+^ concentration was measured under a confocal microscope (LSM 510 Meta, Zeiss; Oberkochen, Germany) by detecting the fluorescence emitted by the Ca^2+^-sensitive indicator Fluo-3 AM. Cells were treated with Fluo-3 AM for 45 min. Culture plates were observed under a 20x objective. The fluorescent signal was detected at an excitation wavelength of 488 nm.

### 2.11. Glucose Uptake Test

C2C12 myocytes were plated at a density of 1 × 10^4^ cells per well in a 96-well culture plate. When the cells had grown to cover more than 80% of the surface of the plate well, the growth medium was changed to a differentiating medium containing 1% FBS, and the cells were then incubated for 4 days to differentiate into myotubes. On the third day of differentiation, 30 *μ*M midodrine was added to the medium of the midodrine-treated group. The myotubes were washed twice with PBS and starved in 100 *μ*L PBS containing 2% (*w*/*v*) bovine serum albumin for 40 min. Myotubes were treated with insulin (100 *μ*mol) and midodrine (30 *μ*M) for 20 min, and then, 2-deoxyglucose was added to the myotubes, which were incubated for 20 min. After three washes with PBS, the myotubes were extracted using extraction buffer. The extracts were centrifuged at 500 rpm for 1–2 min, and then, the supernatants were transferred to fresh tubes. Enzyme mixture, glutathione reductase, substrate, and recycling mixture were added to the diluted extracts along with assay buffer, and the reaction mixture was incubated for 10 min. Optical density was measured at 412 nm.

### 2.12. Oil Red O Staining and Measurement of Hepatic Triglyceride Content

Cells in a 24-well culture plate were fixed in ice-cold methanol for 15 min and then washed with PBS. The cells were stained in Oil Red O solution for 1 h. After washing with 40% isopropyl alcohol for 30 s, PBS washing was performed twice for 5 min each. The cells were observed by optical microscopy and photographed. Next, 1 mL of 100% isopropyl alcohol was added to each well, and the eluted Oil Red O reagent was estimated using an ELISA reader at a wavelength of 530 nm. Triglyceride levels in the liver were measured using a TG quantification kit (Abcam) following the manufacturer's instructions for the fluorometric assay.

### 2.13. Immunohistochemistry

Skeletal muscle tissue slides were prepared from frozen sections. The tissues on the slides were fixed with 4% paraformaldehyde solution for 20 min. The slides were reacted with 0.3% H_2_O_2_ solution for 10 min; after washing, they were blocked with normal serum solution for 1 h. The slides were treated with primary antibody for 1 h and washed with PBS. Secondary antibody was reacted with the slides for 30 min; after washing them with PBS, we reacted premixed VECTASTAIN ABC reagent solution with the slides for 30 min. The slides were then washed with PBS and reacted with DAB substrate solution until color appeared. After washing them with tap water for 5 min, we counterstained the slides with hematoxylin. The slides were then washed with tap water, dried in air, and mounted.

### 2.14. Mitochondrial Staining

A CytoPainter mitochondrial staining kit (ab112143; Abcam) was used according to the manufacturer's instructions for mitochondrial staining of live C2C12 cells. Briefly, CytoPainter was added to live cells and incubated for 1 hour before fixation. Then, stained cells were observed by fluorescence microscopy.

### 2.15. Statistical Analysis

Continuous variables are presented as the mean ± standard error of the mean or standard deviation. Differences between groups were evaluated using the Mann–Whitney *U* test. Overall differences in variables across the 4 groups were analyzed using the Kruskal-Wallis test. BP recordings obtained from the three groups of SHRs from 4 to 8 weeks of age were compared using repeated-measures analysis of variance (ANOVA). All experiments were performed with at least three independent replicates. *p* values < 0.05 were considered to be statistically significant. All statistical analyses were performed using SPSS (ver. 20.0, SPSS Inc.; Chicago, IL, USA).

## 3. Results

### 3.1. Effects of *α*_1_-AR Stimulation on the Expression of Mitochondrial Energetic Molecules, Oxidative Phosphorylation, and Biological Changes in Skeletal Cells and Cells Derived from Other Organs

**Figure 1:**
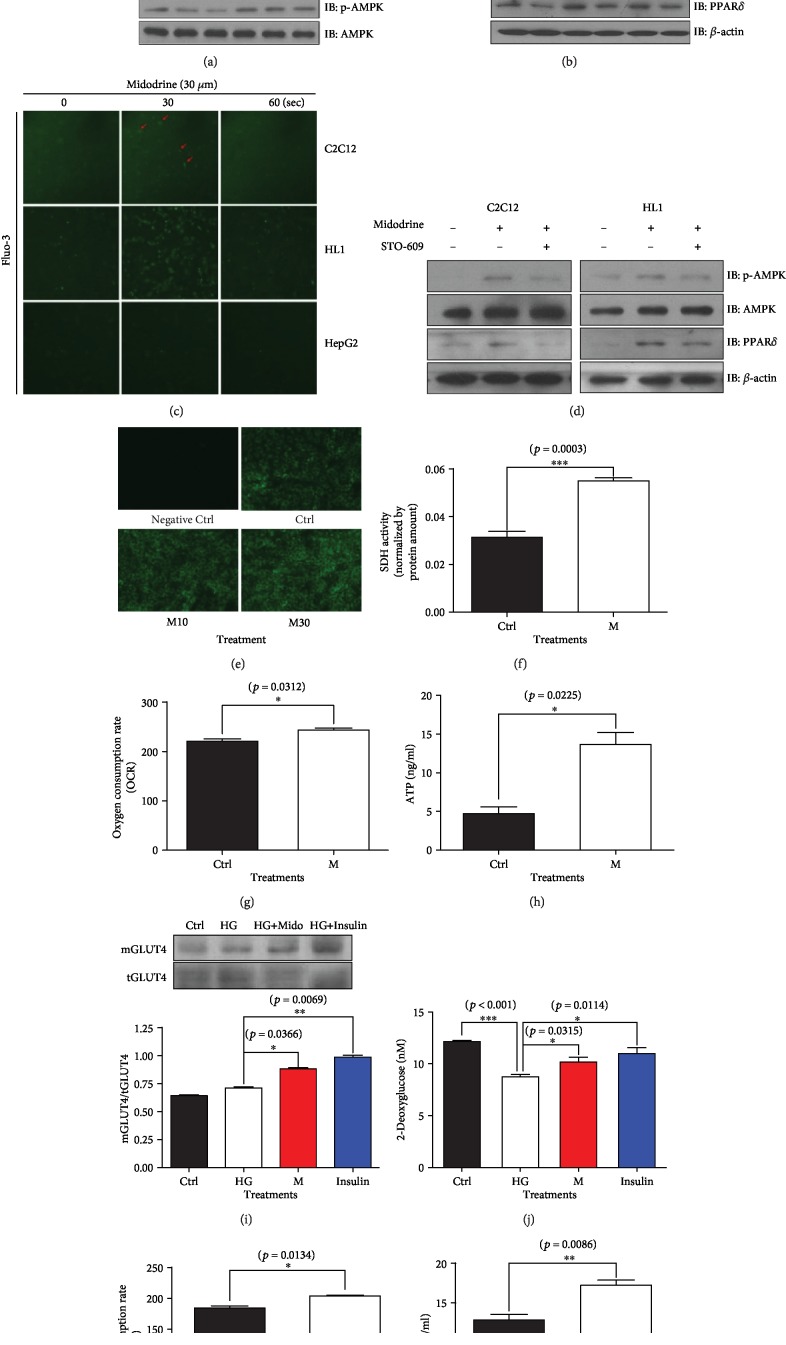
The effects of *α*_1_-AR stimulation on the expression of mitochondrial energetic molecules, oxidative phosphorylation, and biological functions in skeletal and cardiac muscle cells and liver cells. (a, b) The expression of p-AMPK and PPAR*δ* in C2C12, HL1, and HepG2 cells was stimulated with 1–30 *μ*M midodrine for the indicated times. (c) Cytosolic calcium mobilization after midodrine treatment in C2C12 and HL1 cells. Each cell type was pretreated with the calcium reactive dye Fluo-3 AM for 45 min and then stimulated with 30 *μ*M midodrine for the indicated times. Green fluorescence emitted by Fluo-3 AM was detected using confocal microscopy. (d) The phosphorylation of AMPK*α* at Thr172 and expression of PPAR*δ* in C2C12 and HL1 cells after pretreatment with the calcium/calmodulin-dependent protein kinase kinase antagonist STO-609 for 25 min and treatment with midodrine. (e) Fluorescence after using the CytoPainter mitochondrial staining kit in midodrine-treated and control C2C12 cells. Original magnification was 200x. (f) The measured activity of succinate dehydrogenase (SDH) in C2C12 cells. (G) Oxygen consumption rate (OCR) in C2C12 cells treated with midodrine (30 *μ*M), as measured by a Seahorse XFp analyzer. (h) ATP content in C2C12 cells treated with midodrine (30 *μ*M) cultured with low-glucose (5.56 mM) medium. (i) Glucose transporter (GLUT) 4 protein expression in C2C12 cells treated with high glucose (HG) and midodrine (HG+Mido), HG and insulin (HG+Insulin), and the control treatment (Ctrl). (j) The uptake of 2-deoxyglucose in C2C12 skeletal muscle cells treated with midodrine. (k) OCR (measured by the Seahorse XFp analyzer) in H9C2 cells treated with midodrine (30 *μ*M) and cultured with low-glucose (5.56 mM) medium. (l) ATP content in H9C2 cells treated with midodrine (30 *μ*M). Data are expressed as the mean ± standard deviation of triplicate experiments. AMPK: adenosine monophosphate-activated protein kinase; p-AMPK: phosphorylated AMPK; PPAR*δ*: peroxisome proliferator-activated receptor delta; PGC-1*α*: peroxisome proliferator-activated receptor gamma coactivator 1-alpha; mGLUT4: GLUT4 expression of the cell membrane; tGLUT4: total cellular expression of GLUT4; Ctrl: an untreated control group; Mido: midodrine-treated group.

We conducted cell culture experiments to determine whether midodrine, a nonselective *α*_1_-AR agonist, exerts independent effects on PPAR*δ* protein and phosphorylated AMPK (p-AMPK) expression, and we evaluated the mitochondrial oxidative function and ATP production of skeletal muscle cells. We found that a significant increase in p-AMPK expression began to be obtained in C2C12 myocytes following treatment with as little as 3 *μ*M midodrine. PPAR*δ* and p-AMPK expression increased in 3 h, reaching maximum levels approximately 6 h after drug administration (Figures 1(a) and 1(b)), although it is well known that the vascular constrictive effect of midodrine begins to appear within a few minutes [23]. To understand its mechanism of action, we visualized the concentration of intracellular Ca^2+^ using Fluo-3 AM and found that it increased in C2C12 cells, HL1 cells, and HepG2 cells following midodrine treatment (Figure 1(c)). STO-609, a Ca^2+^/calmodulin-dependent protein kinase kinase inhibitor, was used to inhibit calcium signaling. Increases in p-AMPK and PPAR*δ* expression after midodrine treatment were not observed in the presence of STO-609 in C2C12 and HL1 cells (Figure 1(d)). Those results suggest that calcium is involved in midodrine's induction of AMPK phosphorylation and PPAR*δ* expression.

Degree of fluorescence, measured using a CytoPainter mitochondrial staining kit, was higher in the midodrine-treated C2C12 cells than in the control cells (Figure 1(e)), suggesting that midodrine affects mitochondrial concentration. We tested the activity of mitochondrial SDH, a mitochondrial oxidative enzyme involved in both the citric acid cycle and the electron transport chain, and found that the maximal mitochondrial oxidative phosphorylation (measured using OCR estimated with a Seahorse XFp analyzer) and cellular ATP content both increased in C2C12 cells treated with midodrine compared with control C2C12 cells (Figures 1(f)–1(h)).

To investigate the biological effects of *α*_1_-AR on skeletal muscle cells, we evaluated the expression of GLUT4 and the intracellular disposal of 2-deoxyglucose in C2C12 cells treated with midodrine. The GLUT4 expression was higher with midodrine or insulin treatment than with the control treatment (*p* < 0.05; Figure 1(i)). The addition of midodrine or insulin to C2C12 cells also increased the uptake of 2-deoxyglucose (*p* < 0.05; Figure 1(j)). Therefore, midodrine improved insulin sensitivity.

To investigate whether *α*_1_-AR stimulation has the same energetic effects in nonskeletal muscle cells, we conducted the same experiments using cardiomyocytes (HL1 and H9C2 cells) and hepatic cells (HepG2) and obtained the same p-AMPK and PPAR*δ* expression results (Figures 1(a)–1(d)). In H9C2 cells, midodrine increased the maximal OCR (estimated using a Seahorse XFp analyzer) and cellular ATP content (Figures 1(k) and 1(l)).

**Figure 2:**
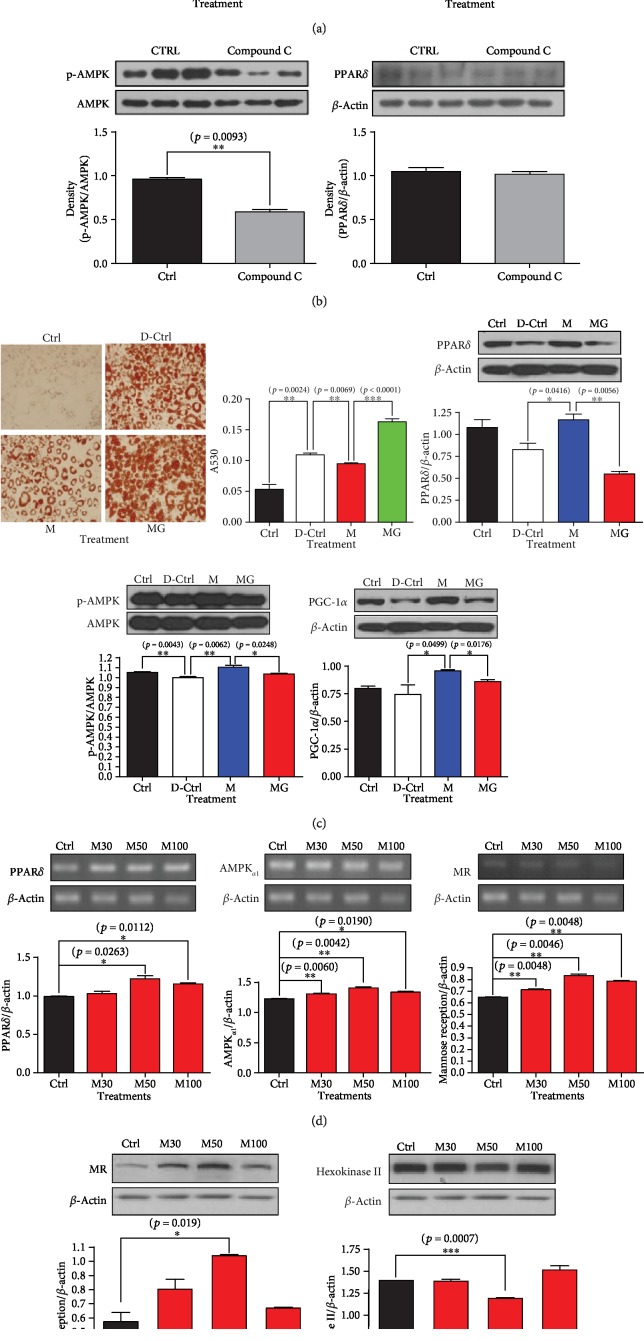
The effect of midodrine on the endothelial expression of p-AMPK and p-eNOS in HUVECs; OCR analyses in H9C2 cells; intracellular fat and the expression of PPAR*δ*, p-AMPK, and PGC-1*α* in differentiated 3T3-L1 cells; and the effects of midodrine on mRNA levels of PPAR*δ*, AMPK*α*_1_, and mannose receptor and protein levels of mannose receptor and hexokinase II in RAW 264.7 macrophage cells treated with different concentrations of midodrine. (a) The expression of phosphorylated AMPK (p-AMPK) and phosphorylated endothelial nitric oxide synthase (p-eNOS) proteins in human umbilical vein endothelial cells (HUVECs) treated with cholesterol and palmitate, and the effects from the addition of GSK0660, a PPAR*δ* antagonist. Ctrl: the control group; CP: the cholesterol- and palmitate-treated group; CPM: the cholesterol-, palmitate-, and midodrine-treated group. (b) The maximal oxygen consumption rate (OCR) analysis as estimated using a Seahorse XFp analyzer and ATP content measured by ELISA in H9C2 cells. (c) The effect of compound C (1 *μ*M) on p-AMPK expression and PPAR*δ* expression. (d) The effect of midodrine on intracellular lipid deposits (Oil Red O staining result) and the protein levels of PPAR*δ*, AMPK, and PGC-1*α* in differentiated 3T3-L1 cells treated with midodrine and GSK0660. (e) The effects of midodrine on mRNA levels of PPAR*δ*, AMPK*α*_1_, and mannose receptor and protein levels of mannose receptor and hexokinase II in RAW 264.7 macrophage cells treated with different concentrations of midodrine. Ctrl: untreated control group; Mido: midodrine-treated group; Mido+GSK0660: midodrine- and GSK0660-treated group.

To investigate whether *α*_1_-AR stimulation has both an energetic effect and cell-specific biological effects in nonskeletal muscle cells, we conducted the same experiments with endothelial cells and adipocytes. Midodrine increased the phosphorylation of both AMPK and eNOS (endothelial nitric oxide synthase) in cultured HUVECs treated with cholesterol and palmitate, and phosphorylation effect was reduced by the addition of GSK0660, a PPAR*δ* antagonist (Figure 2(a)). This result suggests that the energetic regulation caused by *α*_1_-AR stimulation is associated with the regulation of eNOS expression in endothelial cells. In addition, suppressing p-AMPK expression with compound C did not change PPAR*δ* expression (Figure 2(b)). In differentiated 3T3-L1 cells, cellular lipid content was reduced by midodrine treatment, and those reductions were abrogated by the addition of GSK0660 (Figure 2(c)). Corresponding with that result, the protein levels of PPAR*δ*, p-AMPK, and PGC-1*α* increased following midodrine treatment, and those increases were also offset by GSK0660 (Figure 2(c)).

To test for inflammatory activity, we investigated the expression of the mannose receptor, a biomarker of anti-inflammatory M2 macrophages, in RAW 264.7 macrophages. The mRNA and protein levels of the mannose receptor were highest following treatment with midodrine (Figures 2(d) and 2(e)). Additionally, the mRNA expression of PPAR*δ* and AMPK*α*_1_ reached a maximum after treatment with 50 *μ*M midodrine (Figure 2(d)). Reciprocally, the protein level of hexokinase II, a proinflammatory M1 marker, reached a minimum after treatment with 50 *μ*M midodrine (Figure 2(e)).

The results of the cell experiments thus show that pharmacological *α*_1_-AR stimulation directly promoted ATP production by means of mitochondrial oxidative phosphorylation through a PPAR*δ*-AMPK-PCG-1*α* pathway, not only in skeletal muscle cells but also in cardiomyocytes, endothelial cells, hepatocytes, adipocytes, and macrophages. Furthermore, it had relevant cell-specific biological effects, suggesting that *α*_1_-AR causes the metabolic functional changes in cells.

### 3.2. Effects of Long-Term Midodrine Administration on the Expression of Mitochondrial Energetic Molecules and Biochemical Changes in the Skeletal Muscle of Spontaneously Hypertensive Rats

To determine whether the *in vitro* effects of pharmacological stimulation of *α*_1_-AR in various cells extend to an *in vivo* animal model, we used low-dose midodrine treatment in 4-week-old SHRs for 4 weeks without exercise training. We selected SHRs as an animal model for human metabolic syndrome, and 5 weeks of age is a metabolically critical period during which BP and weight increase rapidly.

**Figure 3:**
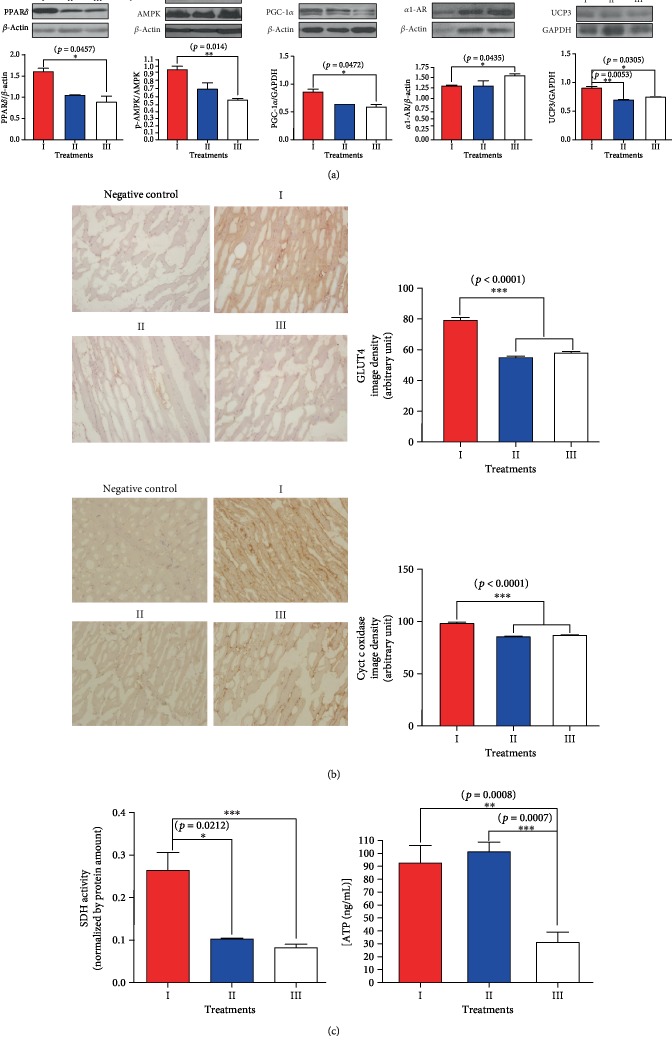
The effect of midodrine on energetic proteins, *α*_1_-adrenergic receptor, UCP3, cytochrome c oxidase, GLUT4, succinate dehydrogenase activity, and ATP concentration in skeletal muscle tissues from SHRs. (a) Midodrine affected the expression of PPAR*δ*, P-AMPK*α*, PGC-1*α*, *α*_1_-adrenergic receptor, and UCP3. (b) Representative examples of immunohistochemical staining for GLUT4 and cytochrome c oxidase in skeletal muscle tissue slides from spontaneously hypertensive rats (SHRs) treated with midodrine or atenolol. Magnification is 200x. Groups: I, midodrine-treated group; II, atenolol-treated group; III, untreated control group. Data are expressed as the mean ± standard error from each group (*n* = 6 per group). (c) Succinate dehydrogenase (SDH) activity in skeletal muscle from SHRs treated with midodrine or atenolol (estimated by a color reaction at 550 nm) and the ATP concentration in skeletal muscle estimated using an enzyme-linked immunosorbent assay.

Consistent with the results from our cell experiments, the midodrine-treated (group I) rats had significantly higher p-AMPK expression in their skeletal muscle than the atenolol-treated (group II) rats and control (group III) animals (*p* < 0.05; Figure 3(a)). PPAR*δ* and PGC-1*α* expression was also elevated in the skeletal muscle of the group I animals compared with the controls (*p* < 0.05; Figure 3(a)). The expression of cytochrome c oxidase, the last enzyme in the respiratory electron transport system in the mitochondrial inner membrane for oxidative fatty acid metabolism, was increased in the skeletal muscle of the group I rats but decreased in the group II and group III animals (Figure 3(b)). SDH activity was higher in the skeletal muscle of the group I rats than in the other groups (*p* < 0.05; Figure 3(c)). Of note, the ATP level in skeletal muscle was far higher in the group I and group II rats than in the group III control rats (*p* < 0.05; Figure 3(c)). The group III SHRs, which had the largest amount of abdominal fat, showed the lowest levels of p-AMPK expression even though they had lower levels of ATP in their skeletal muscle than the midodrine-treated and atenolol-treated animals. The SDH activity in the midodrine-treated rats was much higher than that in the other groups, suggesting that the ATP content in the midodrine-treated rats was associated with increased ATP production in conjunction with AMPK activation, whereas that of the atenolol-treated SHRs was associated with a reduction in ATP consumption rather than increased production in the skeletal muscle.

The expression of uncoupling protein 3 (UCP3) in the skeletal muscle of the group I animals was higher than in the other animals, whereas the *α*_1_-AR expression in skeletal muscle was lower in the group I animals than in the controls (Figure 3(a)). GLUT4 protein expression in the skeletal muscles increased more in the group I rats than in the other animals (Figure 3(b)).

In summary, pharmacological *α*_1_-AR stimulation of skeletal muscle increased the mitochondrial oxidative phosphorylation form of ATP production through the PPAR*δ*-AMPK-PCG-1*α* pathway and changed relevant biological functions in skeletal muscle.

### 3.3. Effects of Long-Term Midodrine Administration on the Expression of Mitochondrial Energetic Molecules and Functional Changes in Multiple Organs in Spontaneously Hypertensive Rats

**Figure 4:**
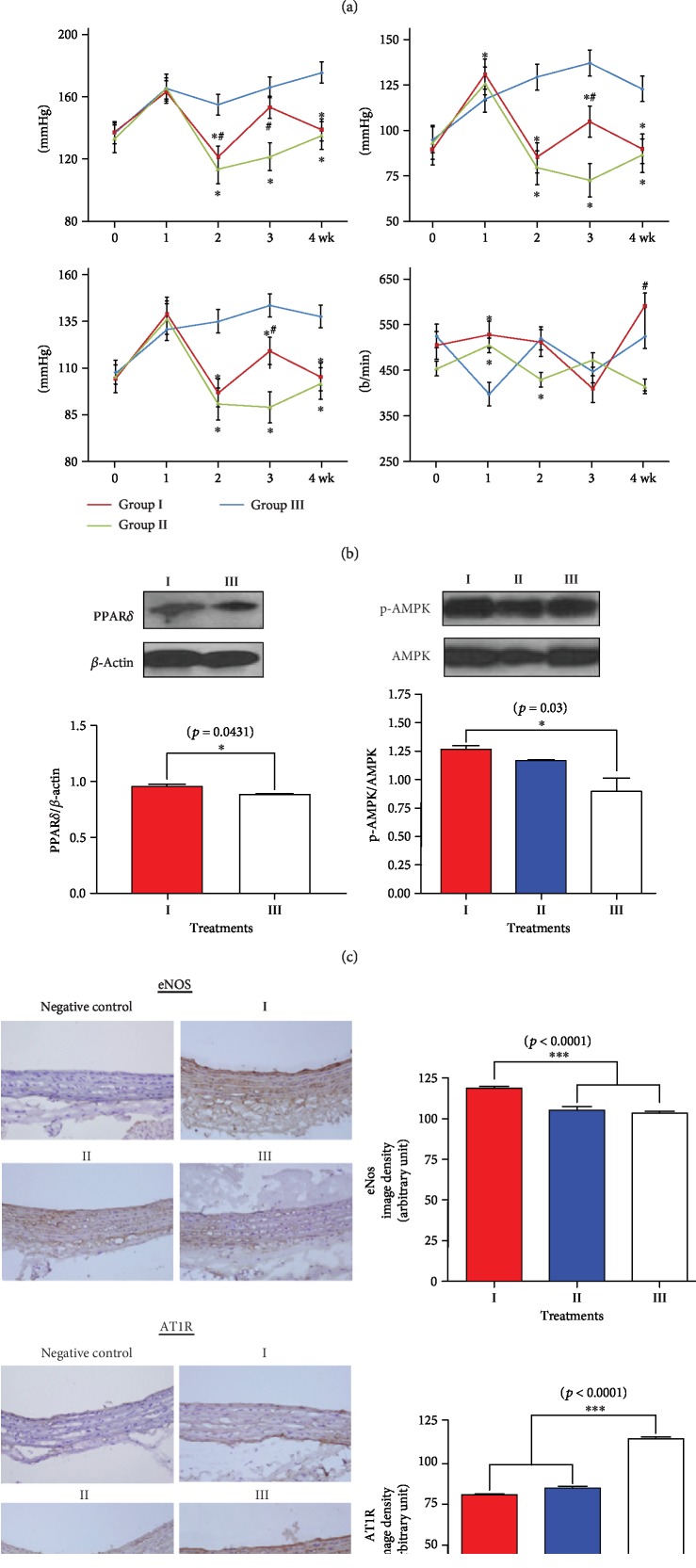
The effect of midodrine on cardiac energetic protein levels, hemodynamic status, aortic eNOS, and AT1R expression in the SHR groups. (a) The cardiac protein levels of p-AMPK, PPAR*δ*, PGC-1*α*, and angiotensin II AT1 receptor; succinate dehydrogenase (SDH) activity; and ATP levels in the midodrine-treated and other animal groups. (b) Systolic, diastolic, and mean blood pressure and heart rate in the spontaneously hypertensive rat (SHR) groups. Data are expressed as the mean ± standard deviation from each group (*n* = 6 per group). ^∗^*p* < 0.05 vs. control; ^#^*p* < 0.05 vs. atenolol. The red, green, and blue lines indicate groups II, III, and IV, respectively. (c) The aortic protein levels of p-AMPK and PPAR*δ* in the midodrine-treated and other SHRs. (d) A representative picture of the immunohistochemistry results for eNOS antibodies in endothelial cells and AT1R expression in medial smooth muscle layers in midodrine-treated and other SHRs. Original magnification is 200 x. Groups: I, midodrine-treated group; II, atenolol-treated group; III, untreated control group. Data are expressed as the mean ± standard error from each group (*n* = 6 per group). eNOS: endothelial nitric oxide synthase; AT1R: angiotensin II AT1 receptor.

We also explored whether pharmacological *α*_1_-AR stimulation alone could activate the expression of energetic molecules and change the function of organs other than skeletal muscle. The midodrine-treated group I animals showed significantly higher p-AMPK, PPAR*δ*, and PGC-1*α* expression and lower expression of the AT1 receptor (AT1R) of angiotensin II in the heart than the control animals (Figure 4(a)). We could not measure cardiac ATP content because of rapid turnover of ATP dynamics in the myocardium.

In cardiac function, the echocardiographic fractional shortening and ejection fraction of the left ventricle in the group I and group II rats were higher than in the group III rats (*p* < 0.05). Both the calculated left ventricular mass and the harvested cardiac weight were smaller in the midodrine-treated animals than in the other groups (Table 1).

Several previous studies reported that *α*_1_-AR stimulation had complex effects on cardiac hypertrophy, and most of those hypertrophic results were not pathologic but physiologic, with preserved or increased contractile function [24]. In a study of transgenic mice with an up to 170-fold increase in cardiac *α*_1_-ARs, myocardial hypertrophy did not develop, but inotropy was markedly enhanced. *α*_1_-AR stimulation apparently switches cardiac failure and hypertrophy into a fetal gene program in which cellular energetics depend more on glycolysis and less on mitochondrial oxidative activity [25]. Therefore, the energetic characteristics of ATP production can explain why pharmacological stimulation of the *α*_1_-AR-PPAR*δ*-AMPK-PGC-1*α* pathway prevents cardiac failure and pathologic hypertrophy, and our results suggest that the mitochondrial oxidative energetic process that was increased in cardiac muscle by midodrine increases cardiac contractility and prevents hypertrophy. This finding supports the result of a previous study that cardiomyocyte-restricted PPAR*δ* deletion perturbs myocardial fatty acid oxidation and leads to cardiomyopathy [6]. Other studies have shown that *α*_1_-AR stimulation is crucial for the maintenance of normal cardiac contractile function, and *α*_1_-AR blocking or knockout exacerbates heart failure, despite lowering the after load [12–14].

Despite immediate BP elevation by *α*_1_-AR-mediated vasoconstriction, midodrine began reducing both systolic and diastolic BP after 1 week of drug administration, and those effects continued until the end of the study (*p* < 0.05, Figure 4(b)). The systolic, diastolic, and mean BPs of the group I and group II rats were lower than those of the control animals (*p* < 0.05) between the 2^nd^ and 4^th^ weeks of the experiment. At the end of the experiment, the mean heart rate of the group II rats was the lowest among the treatment groups. PPAR*δ* and p-AMPK expression in the aorta was higher in the group I animals (Figure 4(c)), and the expression of phosphorylated eNOS protein in the endothelial layer was higher in the group I animals than in the group III controls (Figure 4(d)). The expression of AT1R protein in the aortic medial wall was lower in both the group I and group II rats than in the group III control rats (Figure 4(d)). Regarding the BP-lowering effect of *α*_1_-AR stimulation, our results suggest that *α*_1_-AR has dual biological functions: an early vessel-specific constricting effect [23] and a late vasodilating effect that results from the mitochondrial energetic activation of vascular cells, which reaches its maximum level approximately 6 h after drug administration (Figures 1(a) and 1(b)). This late effect seemed to ameliorate BP elevation as eNOS phosphorylation increased in the endothelial layer and AT1R expression decreased in the medial layer of the artery. These findings support the results of previous studies that *α*_1_-AR mediates eNOS phosphorylation in intact arteries [26] and that the direct activation of AMPK stimulates eNOS expression in arterial endothelial cells [27]. Furthermore, crosstalk has been reported between AMPK activation and AT1R downregulation [28].

**Figure 5:**
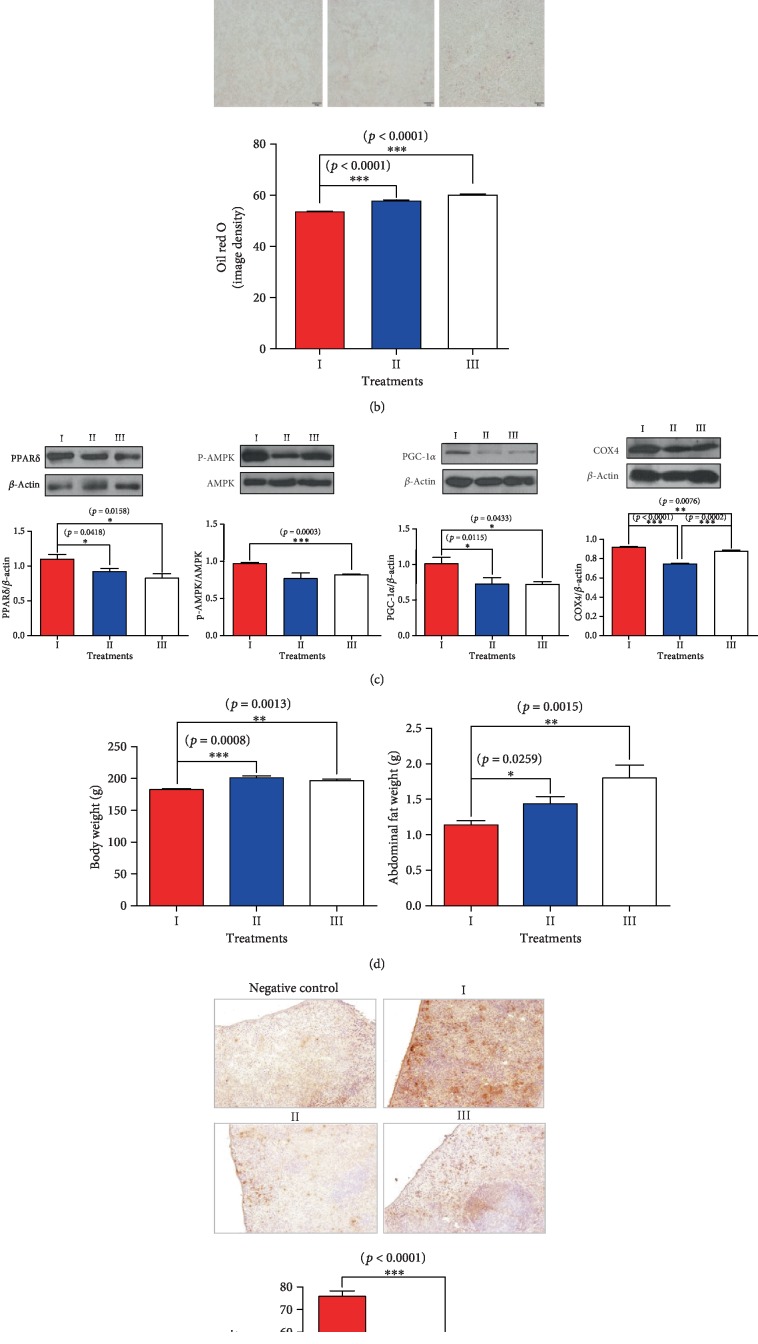
The effect of midodrine on energetic protein expression, HMGCR, and lipid content in liver tissues; cytochrome c oxidase in abdominal fat tissues; body weight and abdominal fat weight; and mannose receptor immunohistochemistry results in spleen tissues from SHRs treated with midodrine or atenolol. (a) The hepatic expression of p-AMPK*α* and HMG CoA reductase (HMGCR) in midodrine-treated and control spontaneously hypertensive rats (SHRs). (b) Lipid accumulation in the liver was measured by Oil Red O staining in the SHR groups. The image density results are expressed as mean ± standard error of the mean. Values were statistically analyzed by the Kruskal-Wallis test or Mann-Whitney test. An upper line on the three bars indicates one-way ANOVA. All experiments were repeated three or more times. Scale bar = 20 *μ*m. (c) The expression of PPAR*δ*, P-AMPK, PGC-1*α*, and COX4 in adipose tissue in SHRs. (d) The body weight and abdominal fat weight differences between the midodrine-treated and other SHRs. (e) A representative picture of the immunochemistry results; the mannose receptor antibody indicated a higher expression of mannose receptors in the midodrine-treated SHRs than in the other rats. Groups: I, midodrine-treated group; II, atenolol-treated group; III, untreated control group. Data are expressed as the mean ± standard error from each group (*n* = 6 per group). COX: cytochrome c oxidase subunit.

In our biochemical study, serum total cholesterol, LDL cholesterol, and HDL cholesterol were lower in the group I and II animals than in the basal and III ones (*p* < 0.05), but the triglyceride levels did not differ among the groups (Table 2). In the liver, midodrine treatment increased p-AMPK expression and lowered the expression of the HMG CoA reductase protein in association with the lower serum LDL cholesterol level and a lower accumulation of lipid content, but the expression of PPAR*δ* and PGC-1*α* did not change following midodrine treatment (Figures 5(a) and 5(b)).

In abdominal fat, the expression levels of cytochrome c oxidase, p-AMPK, PPAR*δ*, and PGC-1*α* levels increased following midodrine treatment (*p* < 0.05; Figure 5(c)). Taken together with the results of our cell culture experiments, these findings suggest the possible transformation of white adipose tissue to beige. The mean body weight and abdominal fat of the group I midodrine-treated rats were the lowest among the groups, and the body weight of the group II atenolol-treated animals was the highest among the groups at 8 weeks of age; the amount of abdominal fat was also lowest in the group I rats (*p* < 0.01; Figure 5(d)).

The serum levels of the proinflammatory cytokines IL-6 and IL-1*β* were lower in the group I animals than in the other groups, but the differences were not significant (Table 3). The level of IL-1*β* was lower in the group I rats than in the group III controls and group II rats, but that difference did not reach significance either (*p* = 0.194). However, TNF-*α* expression was significantly lower in the group I animals than in group II (*p* < 0.01). Group I showed lower ROS levels than groups II and III, but those differences were not significant. The basal control group showed the highest level of adiponectin among the groups, and the group I rats showed a significantly higher level of adiponectin than the group III controls after adjusting for the weight of visceral fat (*p* < 0.05; Table 3). Interestingly, immunohistochemistry testing showed increased expression of the mannose receptor, especially in the cells at the subcapsular portion of the spleen, in the group I animals compared with the group III controls (*p* < 0.01; Figure 5(e)). This anti-inflammatory effect of *α*_1_-AR-PPAR*δ*-AMPK activation is supported by a report that PPAR*δ* is required for the full expression of the effector phenotype of alternatively activated macrophages [29] and a report that exercise training inhibits inflammation in adipose tissue through both the suppression of macrophage infiltration and the acceleration of phenotypic shifting from M1 to M2 macrophages in obese mice [30].

In summary, pharmacological *α*_1_-AR stimulation of multiple organs, including skeletal muscle, increased the mitochondrial oxidative phosphorylation form of ATP production through the PPAR*δ*-AMPK-PCG-1*α* pathway and changed relevant organ-specific biological functions.

## 4. Discussion

In this study, we have shown that *α*_1_-AR activation promoted the activation of the mitochondrial oxidative phosphorylation pathway for ATP production by stimulating mitochondrial energetic molecules (PPAR*δ*, AMPK, and PGC-1*α*) in all representative cells and organs tested, including skeletal muscle, and affected their cell-specific biological functions in both *in vitro* and *in vivo* conditions. Furthermore, the mechanism by which *α*_1_-AR activation stimulates ATP production might work mainly through PPAR*δ*, and a previous study reported that PPAR*δ* functions as an upper regulator for AMPK [31]. Pharmacological *α*_1_-AR stimulation mimicked the changes that occur in skeletal muscle in response to exercise. Surprisingly, *α*_1_-AR stimulation reprogrammed the energetic pathway not only in skeletal muscle but also in other cells and organs; therefore, the physiological effects of *α*_1_-AR are not specific to the exercising muscle. In our *in vivo* tests, the biological functioning of cells and organs other than skeletal muscle changed in ways similar to the healthy effects of exercise, even though the animals did not have exercise training, suggesting a wide distribution of *α*_1_-ARs in multiple organs. We assume that this exercise mimetic effect is associated with the coupling between the cellular fueling metabolism and functional changes because it occurs with only pharmacological *α*_1_-AR stimulation, without exercise or myokine treatment.

Currently, the repetitive contraction and relaxation of skeletal muscle is considered necessary to get the healthy effects of exercise [8]. Metabolically, the basic response to endurance exercise is the production of sufficient ATP through the mitochondrial oxidation of fuels in skeletal muscle. The nutritional consumption required for ATP production is considered to affect whole body metabolism through changes in muscle, which uses 40%–50% of a body's whole energy metabolism, and those changes are enough to produce a healthy exercise effect on the whole body. However, in this study, we have shown that *α*_1_-AR activation in skeletal muscle cells reprograms those cells to increase ATP production by enhancing the mitochondrial oxidative phosphorylation process. Furthermore, it affects biological functions at the individual cellular level with calcium signaling, as seen in exercise. The AMPK phosphorylation that occurs with midodrine treatment is a result of PPAR*δ* activation because the biological effects were abrogated by the addition of a PPAR*δ* antagonist, GSK0660 (Figures 2(a) and 2(c)), and an antagonist for AMPK did not affect PPAR*δ* expression (Figure 2(b)). Because previous studies reported the regulation of metabolic functional change for organ-specific biological functions through the reprogramming of energetic metabolism toward the mitochondrial oxidative phosphorylation pathway in immune cells [4], tumor cells [32], skeletal muscle [8, 9, 33], myocardium [6], and hematopoietic stem cells [7], we also used pharmacological *α*_1_-AR stimulation in the absence of exercise to investigate whether the coupling of cellular fueling metabolism to functional changes works in all cells and organs, and we have indeed shown that the paradigm worked in all the representative cells and organs we tested.

**Figure 6:**
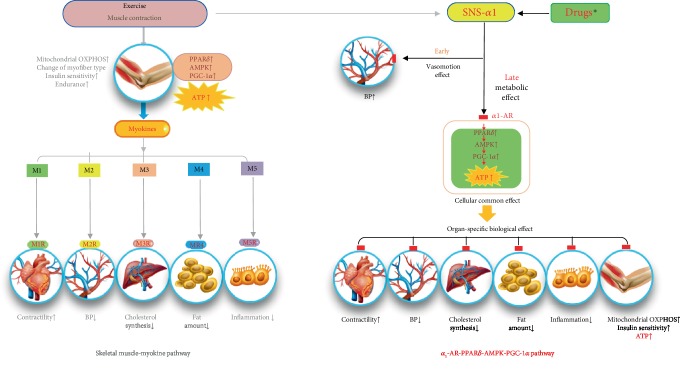
Schematic diagram of our proposed explanation for the multiple organ functions of the *α*_1_-adrenergic receptor agonist midodrine compared with the function of myokine production during exercise. Two presumptive ways have been described to explain how physical exercise produces systemic health benefits: a muscle-derived effect in which exercise activates skeletal muscle energetics through energy consumption and the production of myokines and an *α*_1_-AR-derived effect with downstream transducers that use PPAR*δ*-AMPK-PGC-1*α* signaling to directly activate energetic molecules in all body organs, including the heart, arteries, liver, adipose tissue, and immune cells. Both systems have independent organ crosstalk and network formation. *α*_1_-ANS: *α*_1_-adrenergic nervous system; ↑: increased; ↓: decreased; AR: adrenergic receptor; BP: blood pressure. M1, M2, M3, M4, and M5 and M1R, M2R, M3R, M4R, and M5R represent specific myokines and their receptors derived from exercising skeletal muscles. Drugs^∗^, midodrine.

Thus, the metabolic changes caused by *α*_1_-AR activation are almost the same as those caused by endurance exercise: simultaneously increasing the cellular ATP production pathway through mitochondrial oxidative phosphorylation and controlling the cell-based organ-specific biological functions in multiple organs. Therefore, we suggest that adequate *α*_1_-AR activation is a simple way for a body to endure steady energy-consuming processes during stressful conditions such as exercise or heart failure. A considerable portion of the exercise effect is thus linked to *α*_1_-AR activation independent of skeletal muscle exercise, and the exercise effect attributed to the exercising muscle *per se* could be overestimated in most previous studies of exercise. Although the effect of exercise on muscles seems mainly to be due to the exercise itself, *α*_1_-AR activation in other organs is expected to be the main mechanism of the exercise effect in the whole body. However, the results of this study do not address the organ crosstalk phenomenon caused by myokines derived from exercising muscle [34]. In myokine-driven organ crosstalk, muscle movement is an essential part of obtaining the health effects of exercise. However, in this study, a similar health effect in multiple organs was obtained by administering only a small dose of an *α*_1_-adrenergic. We think that the myokine mechanism and *α*_1_-AR stimulation complement each other in generating the health effects of exercise (Figure 6). We suggest that the total exercise effect results from both the myokine pathway and the PPAR-AMPK-PGC-1*α* pathway driven by *α*_1_-AR stimulation, but we do not yet know the exact proportion that each pathway contributes to the exercise effect. Further studies are needed to clarify that issue. The sequential stimulation of the PPAR-AMPK-PGC-1*α* pathway by midodrine can be proved directly and indirectly, as shown in Figure 2 and the supplementary materials ([Supplementary-material supplementary-material-1]), our previously published article [31], and other reports [35, 36].

In our study, we did not administer a PPAR*δ*-, AMPK-, or PGC-1*α*-agonist to stimulate mitochondrial oxidative phosphorylation. Only an *α*_1_-AR stimulant was used, and interestingly, the expression of all those energetic molecules was increased by *α*_1_-AR activation. Although several studies have shown that some of the exercise-related effects on muscle were obtained with direct stimulation of PPAR*δ*, AMPK, or PGC-1*α*, none of those studies reported on the systemic healthy exercise effect. In addition, previous reports have listed cancer development [37] and increased cardiac risk [38] among the side effects of PPAR*δ*, AMPK, and PGC-1*α* stimulation. Thus, a safe and effective drug to mimic the heathy exercise effect by directly stimulating PPAR*δ*, AMPK, or PGC-1*α* is not yet available for clinical use.

When *α*_1_-AR is activated by midodrine in clinical practice, it is safe, and side effects are rare [39]. Regarding the dose of midodrine required to have a metabolic effect, we found a significant increase in p-AMPK, PPAR*δ*, and PGC-1*α* expression in C2C12 myocytes following treatment with as little as 3 *μ*M midodrine. In the animal study, we administered approximately 0.3 mg/kg/d (1.2 *μ*M/kg/d) of midodrine to the rats, equivalent to approximately 0.05 mg/kg/d in human clinical practice. We administered the daily amount of midodrine at a concentration of 10.0 *μ*M to account for the daily amount of water drunk by an individual rat, and the peak serum concentration of midodrine in the rats was calculated to be about 3.38 *μ*M in a previous pharmacokinetic study [40]. This treatment was enough to stimulate energetic molecules and biological effects in organs and arteries. Therefore, it is likely that the metabolic effect of *α*_1_-AR activation could be obtained in animal experiments at a concentration of midodrine that was even lower than the lowest effective concentration in our cell experiments.

Although selective *α*_1_-AR agonists will play a crucial role in studying how different *α*_1_-AR subtypes are distributed in different organs and how they affect cell-specific effects, we used midodrine, a nonselective *α*_1_-AR agonist that binds to *α*_1_A-, *α*_1_B-, and *α*_1_D-AR [41] in this study. We deemed it suitable for studying integrated healthy exercise effects because it stimulates all the subtypes of *α*_1_-AR, which are allocated throughout the body according to physiological requirements.

Our results here suggest that *α*_1_-AR works through two possible signal transduction pathways. To date, *α*_1_-ARs are understood to signal through G*α*q/11, which leads to the activation of phospholipase C, which causes an increase in inositol phosphate (IP) and diacylglycerol that in turn cause the release of calcium from IP-sensitive stores and the activation of protein kinase C, respectively. That cascade activates various effector enzymes (including PLC, PLA2, and PLD), Ca^2+^ channels, and Na^+^-H^+^ and Na^+^-Ca^2+^ exchange and activates or inhibits K^+^ channels [42]. Additionally, *α*_1_-AR activation could lead to transcriptional activation of early- and late-response genes [17]. In most cells, the primary functional response to the activation of all *α*_1_-AR subtypes is an increase in intracellular Ca^2+^, which involves both early responses such as vascular contraction [17] and late metabolic responses through PPAR*δ*-AMPK-PGC-1*α* activation in vessels and other organs (Figures 2–5). The vessel-specific vasoconstrictive effect appears within 1 hour of midodrine intake, with BP elevation persisting for 2 to 3 hours [43], but the effect of PPAR*δ* and p-AMPK expression increases within 3 h, reaching the maximum level approximately 6 h after drug treatment in the present study (Figures 1(a)–1(c)). Differences in the onset and duration of these two functions presumably indicate that the effects of *α*_1_-AR activation are related to the presence or absence of specific gene expression patterns.

## 5. Conclusion

In conclusion, pharmacological *α*_1_-AR stimulation reprograms cellular energetic metabolism mainly through PPAR*δ* toward the mitochondrial oxidative phosphorylation form of ATP production and consequently regulates biological cell functions, mimicking the healthy effects of exercise; the whole body distribution of *α*_1_-ARs is associated with effects in multiple organs throughout the body.

## Figures and Tables

**Table 1:** M-mode echocardiographic measurements in the midodrine-treated, atenolol-treated, and 8-week-old untreated control groups of rats.

Parameters	Midodrine (group I)	Atenolol (group II)	Control (group III)	*p* value
LV septum in diastole (mm)	1.46 ± 0.13^a^	1.50 ± 0.13^a^	1.45 ± 0.11^a^	0.326
LV posterior wall in diastole (mm)	1.54 ± 0.13^a^	1.67 ± 0.16	1.54 ± 0.15^a^	0.005
LV internal dimension in diastole (mm)	6.06 ± 0.42^a^	6.33 ± 0.35^a^	6.37 ± 0.72^a^	0.108
LV internal dimension in systole (mm)	3.32 ± 0.54^a^	3.43 ± 0.28^a^	3.82 ± 1.01	0.045
LV fractional shortening (%)	45.48 ± 6.25^a^	45.70 ± 5.82^a^	38.77 ± 8.59	0.002
LV ejection fraction (%)	81.55 ± 6.12^a^	82.00 ± 5.31^a^	73.87 ± 10.13	<0.001
LV mass by ASE (g)	1.04 ± 0.06	1.10 ± 0.07^a^	1.07 ± 0.12^a^	0.031
Body weight (g)	238.24 ± 11.69^a^	296.46 ± 16.73^b^	236.20 ± 7.58^a^	0.009

Variables are expressed as mean ± standard deviation. Echocardiography was performed as described in Materials and Methods. LV: left ventricle; ASE: American Society of Echocardiography. The *p* values represent overall differences among groups as determined by the Kruskal-Wallis test. ^a,b^Data values with the same letter did not differ significantly based on the Mann-Whitney test.

**Table 2:** Cholesterol and triglyceride concentrations (mmol/L) in plasma samples from SHRs treated with midodrine or atenolol and control animals.

	Basal control	Midodrine (group I)	Atenolol (group II)	Control (group III)	*p* value
Total cholesterol (mM)	1.69 ± 0.29^a^	0.73 ± 0.15^a^	1.01 ± 0.27^a^	1.66 ± 0.17	<0.001
LDL cholesterol (mM)	0.43 ± 0.06	0.15 ± 0.32^a^	0.15 ± 0.03^a^	0.40 ± 0.03	<0.001
HDL cholesterol (mM)	0.81 ± 0.08	0.049 ± 0.11^a^	0.52 ± 0.04^a^	0.87 ± 0.15	<0.001
Triglycerides (mM)	0.83 ± 0.33	0.43 ± 0.12	0.77 ± 0.33	0.77 ± 0.31	0.092

Variables are expressed as mean ± standard deviation. The *p* values represent overall differences among groups as determined by the Kruskal-Wallis test. ^a,b^Data values with the same letter did not differ significantly based on the Mann-Whitney test. LDL: low-density lipoprotein; HDL: high-density lipoprotein.

**Table 3:** Levels of serum cytokines, reactive oxygen species, lipids, and adiponectin in the midodrine-treated and control groups.

	Basal control	Midodrine (group I)	Atenolol (group II)	Control (group III)	*p* value
IL-1*β* (*μ*g/mL)	0.22 ± 0.20	0.46 ± 0.422	2.02 ± 1.53	1.23 ± 1.45	0.194
IL-6 (*μ*g/mL)	4.50 ± 0.20	4.82 ± 0.49	5.62 ± 1.42	5.48 ± 1.15	0.360
TNF-*α* (*μ*g/mL)	3.60 ± 0.65	2.64 ± 1.64	8.35 ± 1.36*a*^a^	5.36 ± 1.94	0.006
ROS (ng/mL)	186.8 ± 25.7	204.9 ± 27.5	223.1 ± 45.4	254.8 ± 16.8	0.254
Adiponectin^∗^ (ng/mL/g)	74796.6 ± 13898.0^a^	7585.8 ± 182.3^b^	6136.5 ± 574.7^b,c^	5455.8 ± 709.7^c^	<0.001

Variables are expressed as mean ± standard deviation. The *p* values represent overall differences among groups as determined by the Kruskal-Wallis test. ^a,b^Data values with the same letter did not differ significantly based on the Mann-Whitney test. IL: interleukin; ROS: reactive oxygen species; TNF: tumor necrosis factor. ^∗^Serum adiponectin level adjusted for visceral fat weight.

## Data Availability

We can provide the data when there is a request from other researcher. The e-mail address for the data request is as follows: mdhsseo@unitel.co.kr and lyj2333@hanmail.net.

## Supplementary Materials

Supplementary Materials Supplementary Figure 1: ELISA results for PPAR*δ* (A), AMPK (B), and PGC-1*α* (C) in differentiated 3T3-L1 cells treated with 30 *μ*M midodrine and 150 nM GSK0660. Supplementary Figure 2: Oil Red O staining result in differentiated 3T3-L1 cells treated with 30 *μ*M midodrine and 150 nM GSK0660.
