# Supplementary material for: Stimulation of Alpha1-Adrenergic Receptor Ameliorates Cellular Functions of Multiorgans beyond Vasomotion through PPARδ
Source: PPAR Res. 2020 Feb 1;2020:3785137. doi: 10.1155/2020/3785137 (PMC7016472; doi:10.1155/2020/3785137)

Supplementary figure legends

Supplementary Fig. 1 ELISA results for PPARδ (A), AMPK (B), and PGC-1α (C) in differentiated 3T3-L1 cells treated with 30 uM midodrine and 150 nM GSK0660.

The protein concentrations for PPARδ, AMPK, and PGC-1α were decreased in non treated differentiation control groups, however, the lowered levels were increased in midodrine treated groups. But the effects of midodrine were reversed by the treatment of 150 nM GSK0660 (PPARδ antagonist)

The results are expressed as means ± SEM. Values were statistically analyzed by unpaired *t*-test.

Supplementary Fig. 2 Oil Red O staining result in differentiated 3T3-L1 cells treated with 30 uM midodrine and 150 nM GSK0660.

The lipid accumulation was decreased in midodrine treated groups, however, the effect of midodrine was offset by the treatment of 150 nM GSK0660 (PPARδ antagonist). Original magnification is 200 x.

The results are expressed as means ± SEM. Values were statistically analyzed by unpaired *t*-test.

Supplementary Fig. 1

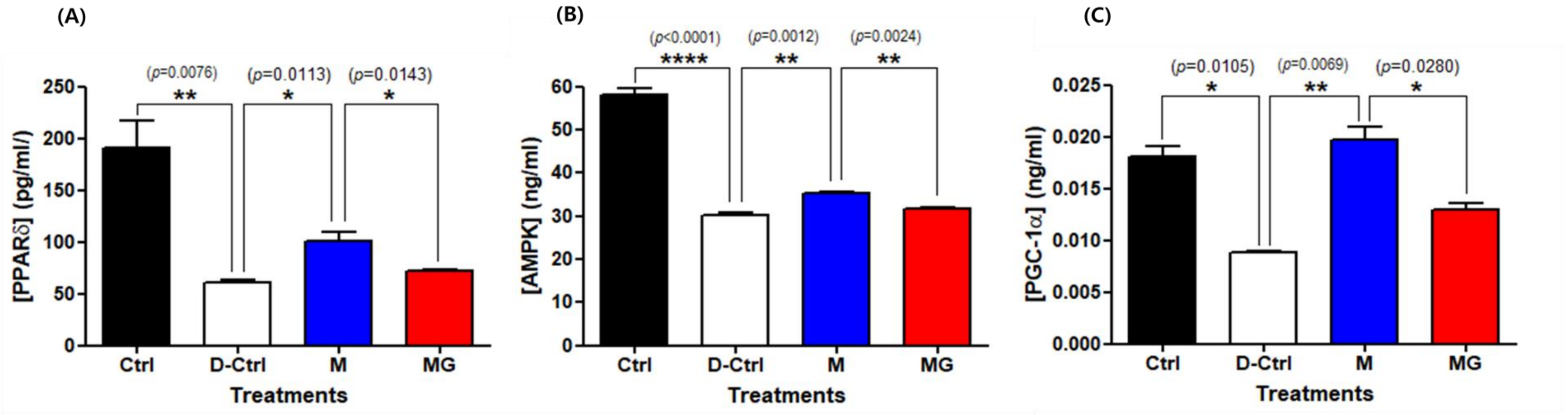

Additional Fig. 2

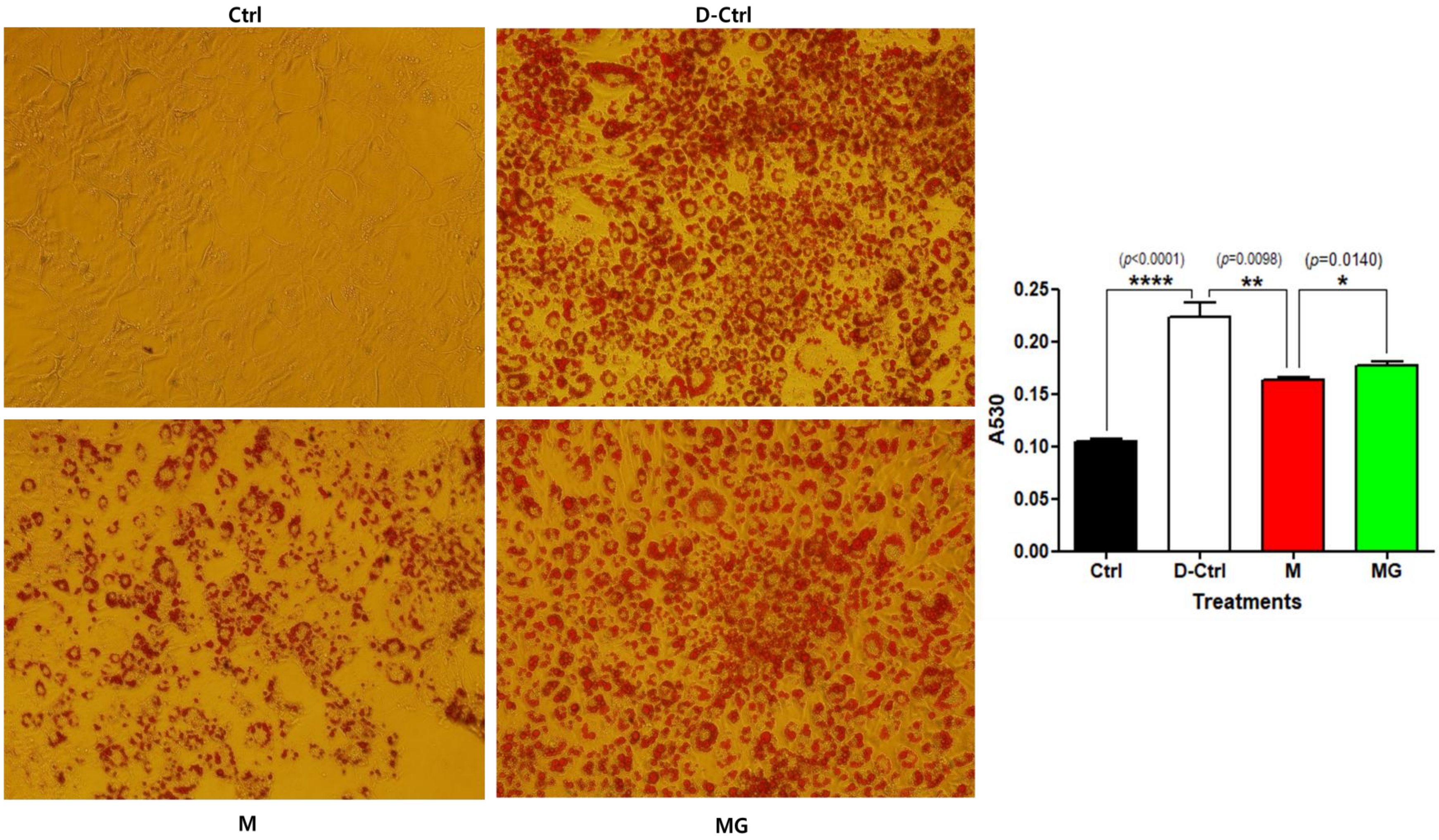

Supplement: Supplementary Materials — Supplementary Figure 1: ELISA results for PPARδ (A), AMPK (B), and PGC-1α (C) in differentiated 3T3-L1 cells treated with 30 μM midodrine and 150 nM GSK0660. Supplementary Figure 2: Oil Red O staining result in differentiated 3T3-L1 cells treated with 30 μM midodrine and 150 nM GSK0660. [file 3785137.f1.pdf]
